# Rapid and sensitive detection of chikungunya virus using one-tube, reverse transcription, semi-nested multi-enzyme isothermal rapid amplification, and lateral flow dipstick assays

**DOI:** 10.1128/jcm.00383-24

**Published:** 2024-08-14

**Authors:** Xinlin Wu, Gaowen Liu, Yingchao Chang, Mengyuan Zheng, Li Liu, Xueshan Xia, Yue Feng

**Affiliations:** 1 Faculty of Life Science and Technology, Kunming University of Science and Technology, Kunming, Yunnan, China; 2 Yunnan Kecan Biotechnology Co., Ltd, Kunming, Yunnan, China; 3 Yunnan Provincial Key Laboratory of Public Health and Biosafety, Kunming Medical University, Kunming, Yunnan, China; The University of North Carolina at Chapel Hill School of Medicine, Chapel Hill, North Carolina, USA

**Keywords:** chikungunya virus, multi-enzyme isothermal rapid amplification (MIRA), lateral flow dipstick (LFD) strips, semi-nested PCR

## Abstract

Chikungunya fever is an acute infectious disease caused by chikungunya virus (CHIKV), which is transmitted by *Aedes* mosquitoes. Simple, rapid, and sensitive detection of CHIKV is critical for its prevention and spread. To address this issue, we combined one-tube, reverse transcription semi-nested, multi-enzyme isothermal rapid amplification, and lateral flow dipstick strips assay to detect CHIKV RNA. The study used a 318-bp gene fragment of CHIKV NSP4 as the target of the assay. This method of amplification takes 30 min for two-step amplification at 39°C. The dilution of amplification products was added to the LFD strip with results visible to the naked eye after 10 min. The method has a sensitivity of 1 copy/μL for the detection of CHIKV RNA, which is 100-fold higher than the conventional reverse transcription-multi-enzyme isothermal rapid amplification and 10-fold higher than the reverse transcription quantitative PCR (RT-qPCR) method. In addition, the method demonstrated good specificity and a better detection rate (85.7%, 18 of 21) than RT-qPCR (80.9%, 17 of 21) in clinically confirmed patient plasma samples. Thus, the rapid CHIKV RNA assay developed in this study will be an important tool for the rapid and accurate screening of patients for chikungunya fever.

**IMPORTANCE:**

This study presents a new one-tube, reverse transcription semi-nested, multi-enzyme isothermal rapid amplification assay combined with lateral flow dipstick strips for the detection of CHIKV. This technique significantly improves sensitivity and outperforms RT-qPCR for the detection of CHIKV, especially in samples with low viral loads. It is also significantly faster than conventional RT-qPCR and does not require special equipment or a standard PCR laboratory. The combination of the isothermal amplification technology developed in this study with point-of-care molecular testing offers the potential for rapid, on-site, low-cost molecular diagnosis of CHIKV.

## INTRODUCTION

Chikungunya virus (CHIKV) is a pathogen that can be transmitted to humans by *Aedes* mosquitoes, causing debilitating and long-lasting arthritis (1). Symptoms of chikungunya typically include sudden onset of high fever (39°C–40°C), maculopapular rash, and severe arthralgia during the acute phase of infection (2). Large outbreaks of the virus have been reported in Southeast Asian countries such as Thailand and Myanmar, with more than 27,000 confirmed cases (3). In China, local epidemics caused by imported cases have been reported in Yunnan, Guangdong, and Zhejiang provinces (4–7). Additionally, recent reports have indicated that there have been outbreaks of chikungunya in the Americas in recent years. In the first 4 months of 2023 alone, over 214,000 cases were reported (8). Although there is currently no specific anti-viral drug for the treatment of Chikungunya virus infection, the development of a specific and sensitive test for CHIKV is essential for the selection of an effective treatment regimen and the rapid implementation of environmental controls to prevent the spread of the CHIKV.

Currently, there are two main methods for diagnosing CHIKV infection. These are nucleic acid-based detection [reverse transcription PCR (RT-PCR) and reverse transcription quantitative PCR (RT-qPCR)] and serological detection. Previous studies have developed several quantitative PCR (qPCR) assays for different CHIKV genes, such as NSP1 and NSP2 (9, 10). However, the sensitivity of these assays is low, and the cycle threshold (Ct) cutoff for a positive is between 35-37. It is well known that all amplification techniques have a plateau period, especially for samples with cycle threshold values between 35 and 40, which are difficult to define as negative or positive by qPCR. In clinical testing, samples in this “gray area” are the most challenging and require duplicate testing, resulting in wasted labor and material resources. In addition, loop-mediated isothermal amplification (LAMP) has also been used for CHIKV detection, and the sensitivity of the developed RT-LAMP assay is only 163 RNA copies/μL (11). This sensitivity is insufficient for clinical detection.

It is important to develop a convenient and susceptible diagnostic technique for the early detection of CHIKV RNA. In recent years, the rapid development of new-generation point-of-care molecular testing (POCT), particularly isothermal amplification technology such as recombinase polymerase amplification (RPA) (12) and multi-enzyme isothermal rapid amplification (MIRA), which is characterized by simplicity, sensitivity, and accuracy, has been widely used for the rapid diagnosis of infectious diseases (13, 14). MIRA, which uses a recombinant enzyme from *Streptomyces azureus* instead of T4 UvsX, has better amplification efficiency, stability, and resistance to interference than RPA (14). Therefore, it is an excellent diagnostic technique for infectious diseases and is gaining popularity.

**Fig 1:**
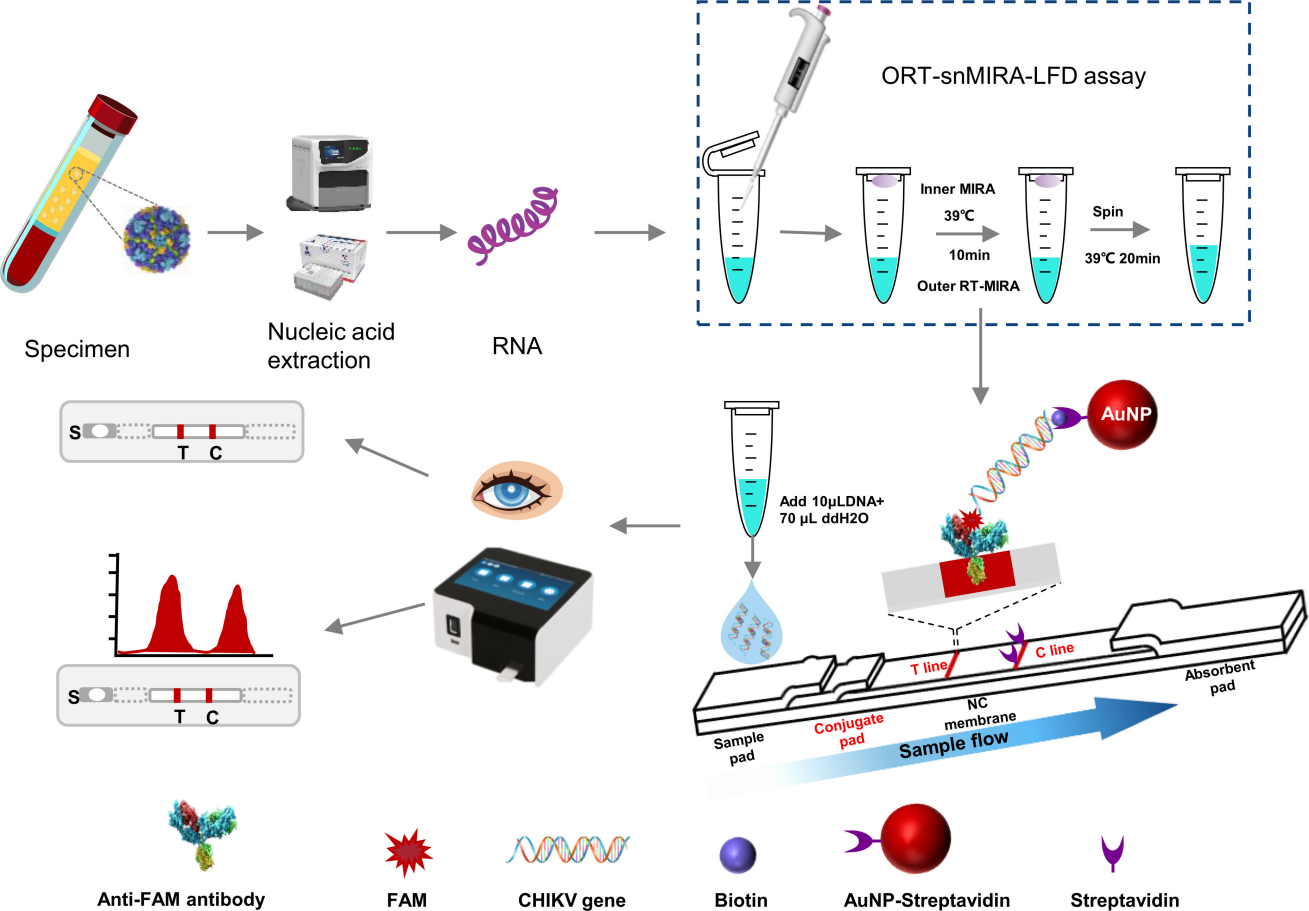
Detection and identification of CHIKV by one-tube, reverse transcription, semi-nested multi-enzyme isothermal rapid amplification, and lateral flow dipstick (ORT-snMIRA-LFD) assay. Schematic diagram of ORT-snMIR-LFD workflow. CHIKV plasma samples were automatically extracted (about 20 min) to obtain viral RNA. Viral RNA was put into the ORT-snMIRA -LFD assay, which was first performed by outer reverse transcription multi-enzyme isothermal rapid amplification (RT-MIRA) to reverse transcribe the viral RNA into dsDNA and pre-amplified by MIRA. Subsequently, the inner MIRA located in the cap of the tube entered into the bottom of the tube under the centrifugal force to perform the next round of MIRA amplification (30 min). At the end of the MIRA reaction, the target DNA is heavily enriched and forms a DNA complex, which is 5′ labeled with fluorescein amidite (FAM) fluorescence and 3′ labeled with biotin. This can be detected visually by lateral flow strips or by a gold standard reader. In the positive test result, a positive band is visible to the naked eye at the detection line (T line) because the FAM-dsDNA-Biotin complex in the amplification product is captured by the anti-FAM antibody and the gold nanoparticle (AuNP)-streptavidin binds to the biotin with high efficiency, while the excess AuNPs-streptavidin is captured by the biotin, forming the control line (C line).

In this study, we developed a new method called one-tube, reverse transcription semi-nested, multi-enzyme isothermal rapid amplification, and lateral flow dipstick (ORT-snMIRA-LFD) assay to improve the specificity and sensitivity of isothermal amplification. We used colloidal gold lateral flow dipstick (LFD) strips to detect and characterize CHIKV with a high signal-to-noise ratio (Fig. 1). Our method is significantly faster than conventional RT-qPCR and does not require special equipment or a standard PCR laboratory. The proposed assay is reliable, cost-effective, and convenient for rapid detection of the chikungunya virus in community hospitals, customs, and airports.

## MATERIALS AND METHODS

### Strain and nucleic acid extraction

A detailed description of the pathogens used in this study is provided in Table S1. The different CHIKV strains were obtained by cell culture in a BSL-III laboratory and subjected to a plaque-forming unit (PFU) assay. The DNA and RNA of viruses, bacteria, and fungi were extracted using the QIAamp Viral Mini Kit (QIAGEN, Hilden, Germany).

### Design of the MIRA primer and probe

To identify the optimal test target for CHIKV, we conducted a comprehensive analysis of 1,000 full-length CHIKV sequences available in the GenBank database. A specific MIRA probe was initially designed (Fig. S1). Then four forward primers and two reverse primers were selected before and after the probe position, targeting the highly conserved region of the CHIKV NSP4 gene. The designs of primers and probes were conducted using the Premier version 5.0 software, following the specifications of AMP-Future Biotech Co. Ltd. (Weifang, China). The sequence of primers and probes is presented in Table S2. All the oligonucleotides were synthesized by Taihe Biotech Co., Ltd. (Beijing, China).

### CHIKV RNA standards

RNA transcripts of the partial NSP2-NSP4 gene of CHIKV were obtained by *in vitro* transcription and used as RNA standards. *In vitro* transcription was performed using the T7 High Yield RNA Transcription Kit (Vazyme, Nanjing, China) according to the manufacturer’s instructions. Subsequently, the RNA was quantified using the Qubit version 4.0 instrument (Thermo Fisher Scientific, Waltham, USA).

### RT-qPCR

For quantitative detection of CHIKV RNA, NSP2 was used as a target according to a previously reported method (9). Real-time RT-PCR assays were performed using the Evo M-MLV One-Step RT-qPCR Kit (Accurate, Hunan, China). The 20-µL system contained 10-µL 2 × One-Step RT-qPCR Buffer, 0.4-µL Pro Taq HS DNA Polymerase (5 U/µL), 0.4-µL Evo M, 1 µL each of primer (10 µM) and probe (5 µM). Additionally, 4.2-µL ddH_2_O and 2-µL RNA were added as templates. RT-qPCR reactions were performed on a Gentier 96E instrument (Tianlong, Xi'an, China) according to the described protocol. The process included reverse transcription at 42°C for 5 min, followed by activation at 95°C for 30 seconds, and then 45 cycles of amplification at 95°C for 5 seconds and 60°C for 30 seconds. Finally, the reactions were analyzed using the Gentier-96E software.

### RT-MIRA-LFD assay

The reverse transcription multi-enzyme isothermal rapid amplification (RT-MIRA) assay was performed using the RNA Isothermal Rapid Amplification Kit (AMP-Future, Shandong, China) according to the manufacturer’s instructions. The 50-µL reaction mixture consisted of 29.4-µL A buffer, 8.5-µL ddH_2_O, 2.0-µL primer (10 µM), 0.6-µL specific probe (10 µM), 2.5-µL B buffer, and 5.0-µL RNA template. The reaction was conducted in a metal bath at 39°C for 20 min. Subsequently, 10 µL of the amplification product was collected and added to 70 µL of ddH_2_O, mixed, and then added to colloidal gold LFD strips (AMP-Future). After 10 min, the assay was performed using a colloidal gold immunochromatographic analyzer (Fenxi, Shenzhen, China). The results of the samples were determined based on the Dr value obtained from the optical sensor. The Dr value is calculated as Dr = (T_Area) / (C_Area), where T_Area is the area of the peak corresponding to the T line, and C_Area is the area of the peak corresponding to the C line on the signal curve.

### ORT-snMIRA-LFD assay

The ORT-snMIRA-LFD assay is a two-step process involving outer RT-MIRA and inner MIRA-LFD. In the first step, the RNA Isothermal Amplification Basic Kit (AMP-Future) is used to generate cDNA and pre-amplify the products. The second step is the internal MIRA using the DNA Isothermal Rapid Amplification Kit (AMP-Future). In this step, cDNA is amplified and a colloidal gold strip assay is performed. To perform the assay, the Outer RT-MIRA System (20 µL) and the Inner MIRA System (25 µL) are prepared according to the manufacturer’s instructions. The outer RT-MIRA System is in the bottom of the test tube and the inner MIRA System is on the cap. First, 5 µL of RNA is added to the bottom of the tube for outer RT-MIRA amplification, which is performed at 39°C for 10 min. Next, the inner MIRA system is spun down and incubated at 39°C for 20 min. Finally, 10 µL of ORT-snMIRA product is combined with 70 µL of water and added to colloidal gold LFD strips for detection.

### Speciﬁcity evaluation

A total of 28 pathogens were used, including 16 viruses, 5 bloodstream infectious bacteria, 3 bloodstream infectious yeasts, and 4 infectious skin fungi, for cross-reactivity testing (Table S1). Additionally, three different CHIKV strains representing all known three genotypes,

Asian, West African, and Eastern/Central/South African, were tested.

### Sensitivity evaluation

The limit of detection for the ORT-snMIRA-LFD assay was determined using serial 10-fold dilutions of CHIKV standard RNA ranging from 1 × 10⁶ copies/μL to 1 copy/μL, and viral RNA was extracted from CHIKV cell culture strains ranging from 1 × 10⁵ PFU/mL to 1 PFU/mL. RT-qPCR was simultaneously performed on the above positive samples. This experiment was repeated three times.

### Evaluation of interfering substances

The CHIKV virus was inactivated and then diluted to a concentration of 1 × 10² PFU/mL using negative serum samples. These samples were then mixed with eight potential interfering substances, including ribavirin (an anti-viral), acetaminophen (an antipyretic and analgesic), amoxicillin (an antibiotic), dexamethasone (a hormonal medication), heparin, ethylenediamine tetraacetic acid, sodium citrate, and albumin. Each interfering substance was tested at high, medium, and low concentrations. Subsequently, the ORT-snMIRA-LFD assay was performed on these samples to assess its ability to detect CHIKV in the presence of these potential interfering substances.

### Specimen stability determination

Two sets of quality control specimens were prepared to test the CHIKV assay. The first set contained high positive samples at a concentration of 1 × 10^5^ PFU/mL, while the second set contained low positive samples at a concentration of 1 × 10^2^ PFU/mL. These samples were prepared by mixing cell-cultured inactivated CHIKV strains with fresh negative serum samples. Following preparation, the samples were stored at three distinct temperatures (24°C, 4°C, and −20°C) and then subjected to three replicates of the ORT-snMIRA-LFD assay at 1-, 3-, and 5-day intervals. Furthermore, 1 × 10^2^ PFU/mL specimen was evaluated after three freeze-thaw cycles.

### Clinical samples

In this study, we examined 329 suspected plasma samples from dengue-endemic areas. We used both RT-qPCR and IgM methods to screen for CHIKV according to previously established criteria to confirm the diagnosis of CHIKV (15). We then selected clinically confirmed CHIKV-positive samples and representative negative samples to evaluate the performance of the ORT-snMIRA-LFD assay.

## RESULTS

### Selection of ORT-snMIRA-LFD assay

**Fig 2:**
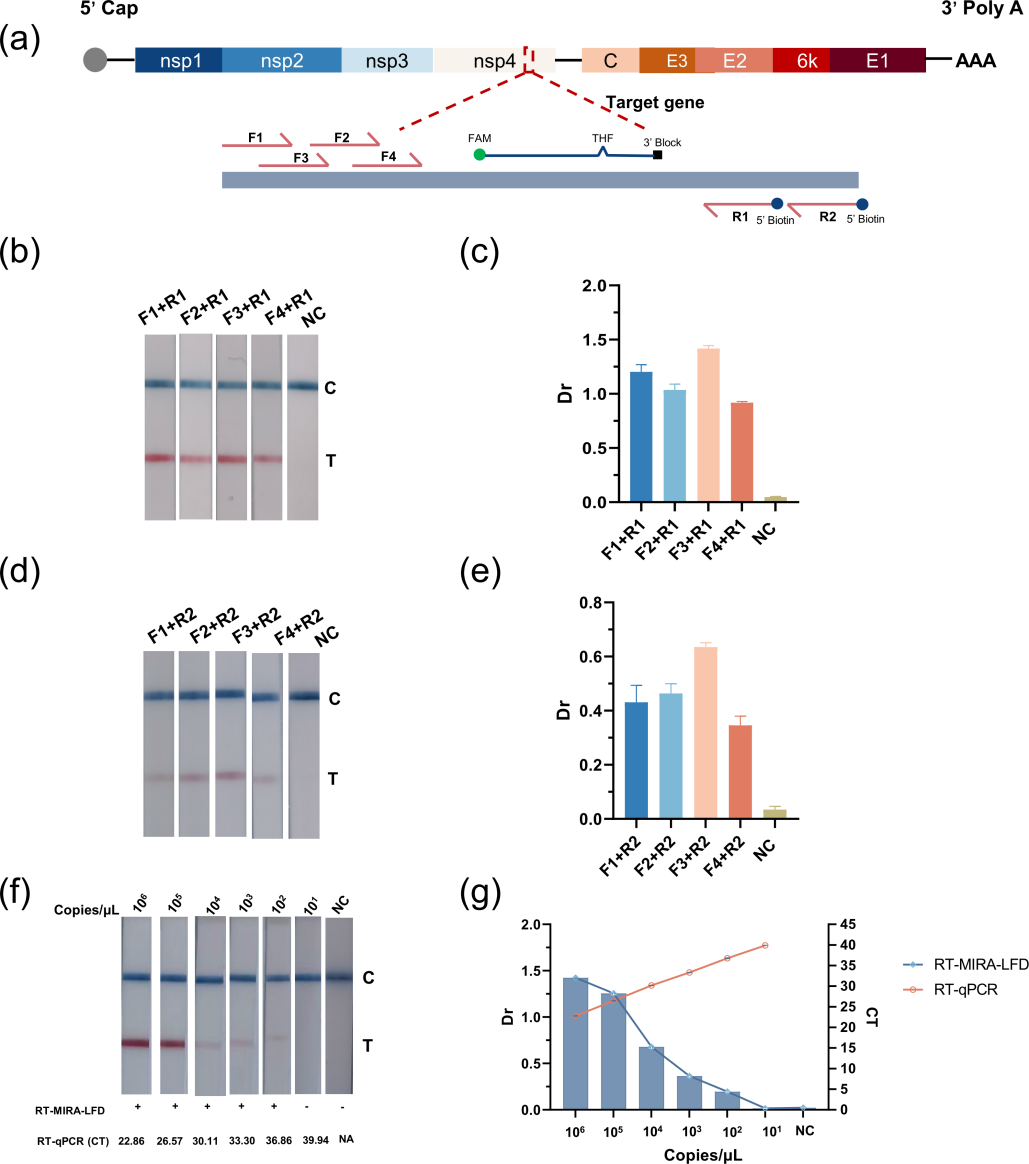
Primer screening and RT-MIRA-LFD reaction system establishment. (a) Schematic diagram of the chikungunya virus genome and the location of the primer probe in the target gene NSP4. Primers were screened using CHIKV RNA with a concentration of 1 × 10^5^ copies/μL as a template. (b and c) Results of reverse primer R1 with forward primer (F1–F4) assay. (d and e) Results of reverse primer R2 with forward primer (F1–F4) assay. (f and g) Comparison of RT-qPCR and RT-MIRA-LFD assay sensitivity using serially diluted CHIKV RNA standards. The Dr values in all assays were obtained by colloidal gold immunochromatographic analyzer: Dr = (T_Area) / (C_Area). The experiment was carried out three times.

The amplification efficiency of different primer combinations was evaluated for four forward primers and two reverse primers that were designed (Table S2). A positive control sample of 1 × 10⁵ copies/μL CHIKV RNA was utilized and amplified under this condition: 39°C for 20 min. The results indicated that the F3/R1 combination exhibited the most robust bands with the highest Dr value, followed by F1/R1 (Fig. 2a through E). Furthermore, the study demonstrated that the lower detection limit of RT-MIRA-LFD using the F3/R1 combination was 100 copies/μL, which is 10-fold lower than that of RT-qPCR (Fig. 2F through G). However, the performance of RT-MIRA-LFD in terms of detection did not meet the desired standards, indicating the need for further optimization. To enhance the sensitivity of the method, the study proposed a novel approach for ORT-snMIRA-LFD assay for the detection of CHIKV RNA. This approach employs the primer combination F1/F3 and R1, which amplifies a 318-bp gene fragment of CHIKV NSP4.

### Optimization of reaction conditions for ORT-snMIRA-LFD assay

**Fig 3:**
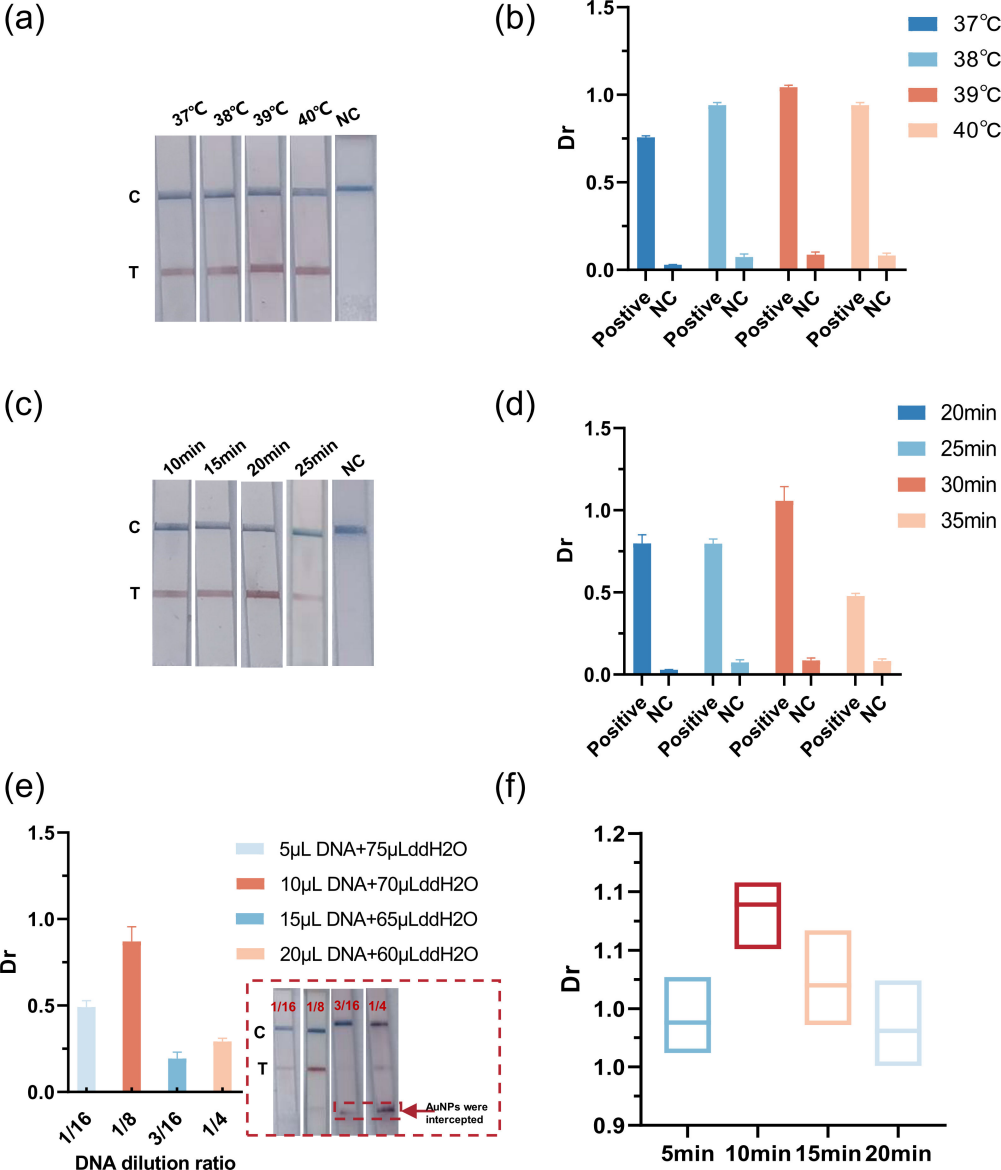
Optimization of reaction conditions for ORT-snMIRA-LFD assay. (a and b) ORT-snMIRA-LFD assay suitable reaction temperatures were determined. (c and d) In the ORT-snMIRA-LFD assay, the reaction time of outer RT-MIRA was fixed at 10 min, and the optimal reaction time of inner MIRA was subsequently optimized. (e) Determination of DNA dilution ratios for detection in colloidal gold LFD strips. (f) Determining the appropriate detection time for colloidal gold LFD strips. The experiment was carried out three times. Dr = (T_Area) / (C_Area). NC, negative control.

A series of parameters were examined to optimize the assay, including reaction temperature, reaction time, DNA dilution ratio in LFD strips, and detection time of LFD strips. These conclusions were reached by evaluating the intensity of the T-line color on the LFD strips and the Dr value. The results indicate that the optimal reaction temperature for ORT-snMIRA is 39°C (Fig. 3a and b), with an optimal reaction time of 30 min (Fig. 3c and d). Moreover, the optimal DNA dilution ratio for the LFD strips is 1:8 (10-μL DNA + 70 µL ddH_2_O) (Fig. 3e). Finally, our findings indicate that the optimal detection time for LFD strips is 10 min (Fig. 3f).

### Specificity of the ORT-snMIRA-LFD assay

**Fig 4:**
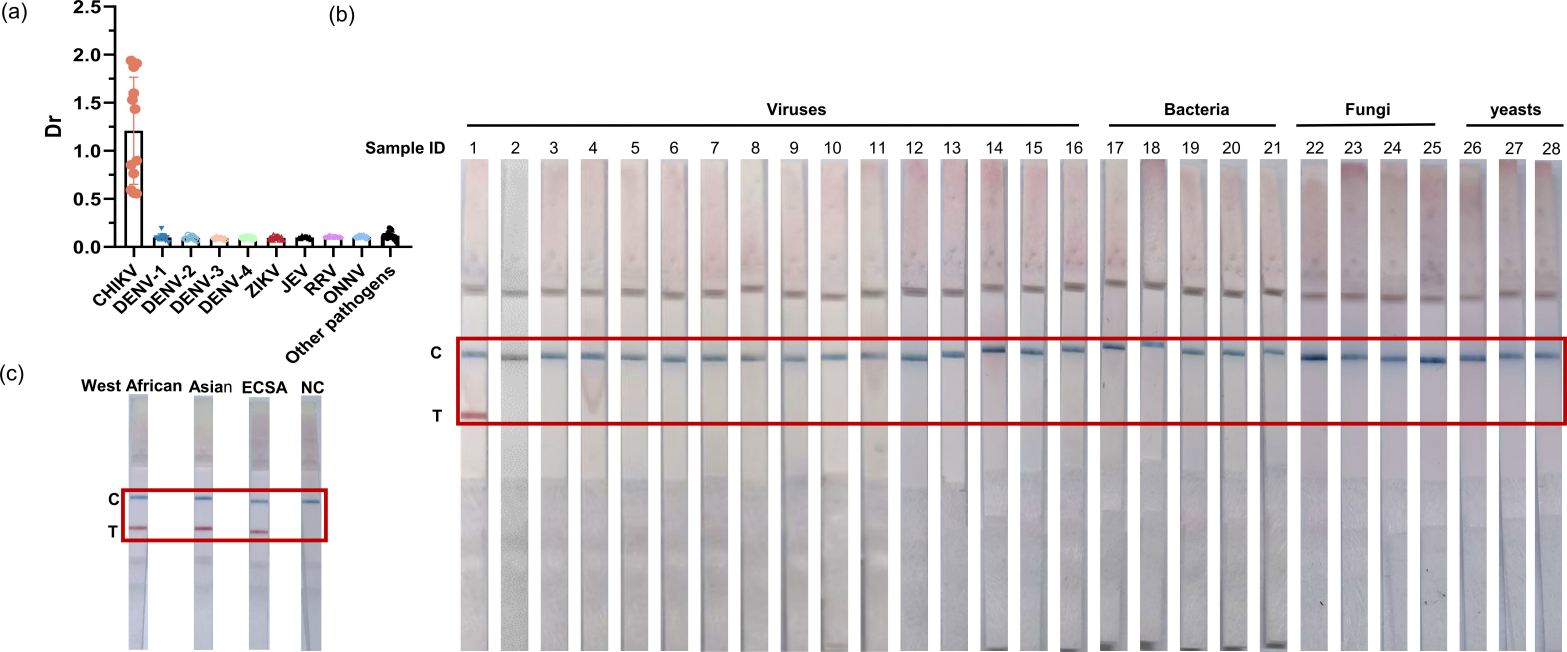
Specificity of the ORT-snMIRA-LFD assay. (a and b) In this study, a total of 28 pathogens were used, including 16 viruses [Zika virus (ZIKV), dengue virus types 1–4 (DENV-1 to -4), Japanese encephalitis virus (JEV), Ross River virus (RRV), O’nyong-nyong virus (ONNV), human immunodeficiency virus type 1, hepatitis B virus, hepatitis C virus, cytomegalovirus, Epstein-Barr virus, adenovirus, and BK virus], five bloodstream infectious bacteria (*Escherichia coli, Pseudomonas aeruginosa*, *Staphylococcus aureus*, *Klebsiella pneumoniae*, and *Acinetobacter baumannii*), three bloodstream infectious yeasts (*Candida albicans*, *Candida glabrata*, and *Candida parapsilosis*), and four skin infectious fungi (*Microsporum gypseum*, *Trichophyton rubrum*, *Malassezia furfur*, and *Trichophyton mentagrophytes*). (c) Three different CHIKV strains were tested, representing all three known genotypes: Asian, West African, and Eastern/Central/South African (ECSA).

To evaluate the specificity of the method, 28 pathogens were tested using the ORT-snMIRA-LFD assay developed in this study. The results showed that only the CHIKV samples tested positive, confirming the high specificity of the technique (Fig. 4; Table S1). In addition, the eight potential interfering substances did not affect the detection of the developed ORT-snMIRA-LFD assay (Fig. S3; Table S3).

### Sensitivity of the ORT-snMIRA-LFD assay

**Fig 5:**
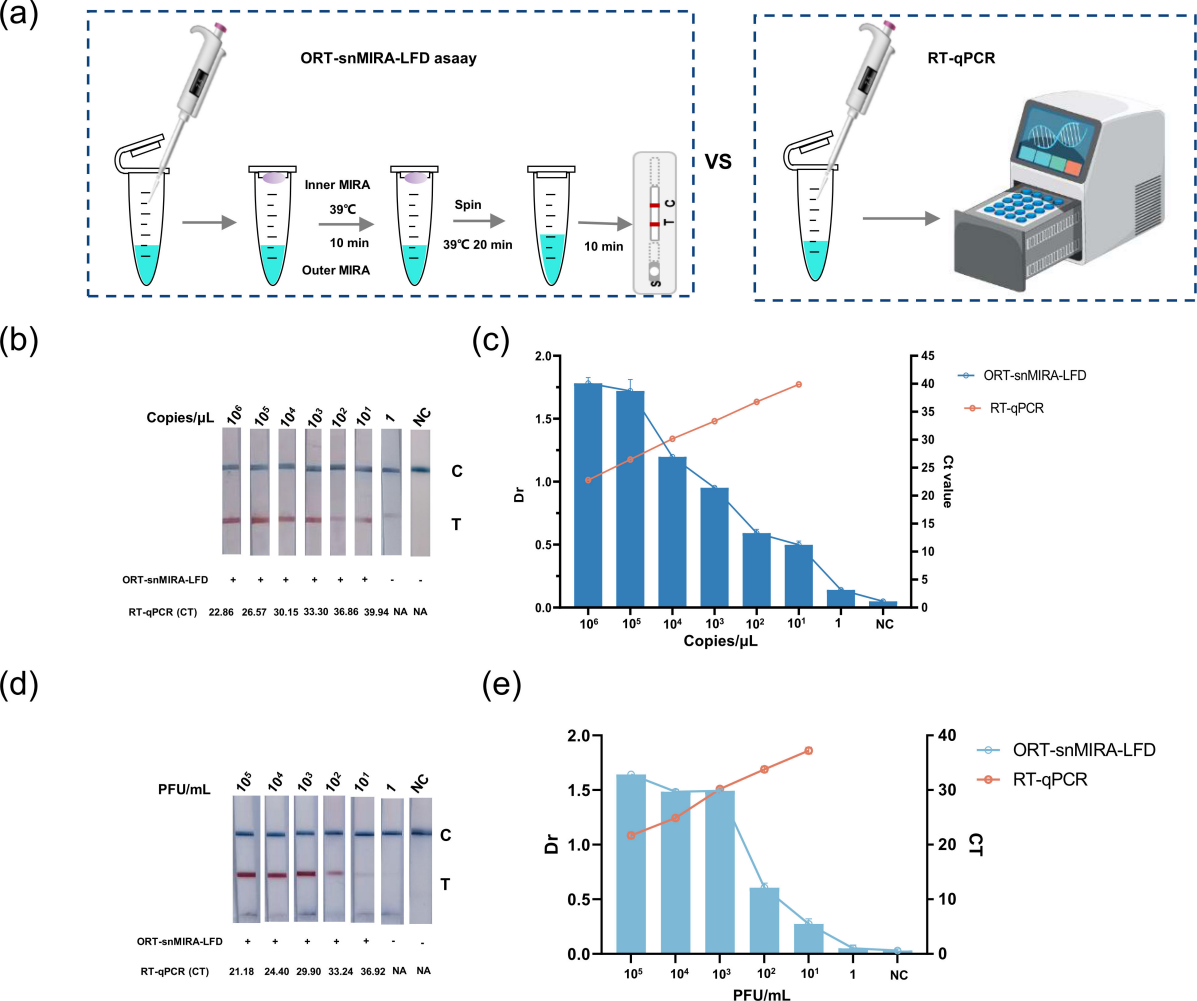
Sensitivity of the ORT-snMIRA-LFD assay. (a) Scheme of the ORT-snMIRA-LFD assay and conventional RT-qPCR. (b and c) Comparison of the sensitivity of ORT-snMIRA-LFD assay and conventional RT-qPCR assays. Serially diluted CHIKV RNA standards (1 × 10^6^ – 1 genome copies/μL) were used for the assay. (d and e) Detection of viral RNA extracted from CHIKV cell cultures ranging from 1 × 10⁵ PFU/mL to 1 PFU/mL using ORT-snMIRA-LFD assay to assess the ability of the assay in detecting natural targets.

The results consistently showed that the developed ORT-snMIRA-LFD assay has a detection limit of 1 copy/μL (Fig. 5b and c) and 10 PFU/mL for detecting CHIKV (Fig. 5d and e; Fig. 2). Furthermore, compared to conventional RT-MIRA, the method showed a 100-fold improvement in detection limit results, but compared to RT-PCR, it showed a 10-fold improvement, indicating higher sensitivity (Fig. 5).

### Evaluation of sample stability

The test results indicate that the samples were not adversely affected by storage at 24°C, 4°C, and −20°C for 1, 3, and 5 days, respectively. Furthermore, the presence or absence of EDTA anti-coagulant in the blood sample did not influence the test results (Table S4).

### Detection of clinical samples

**Fig 6:**
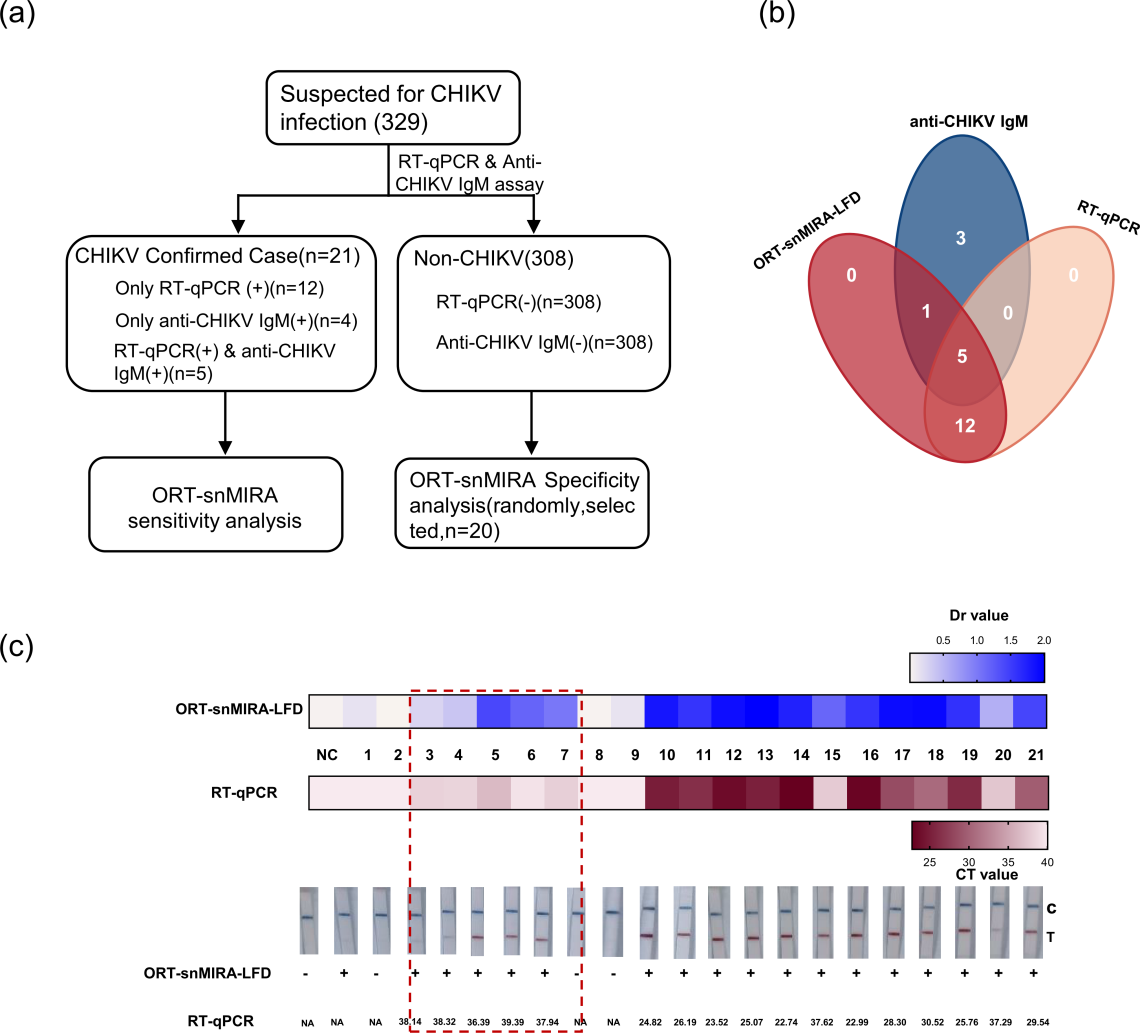
Validation of the ORT-snMIRA-LFD assay in clinical specimens. (a) Clinical study design; 329 suspected patients were enrolled and 21 patients were diagnosed with RT-qPCR and anti-CHIKV IgM. (b) Venn diagram showing the overlap of CHIKV diagnostic test results obtained from ORT-snMIRA-LFD assay, RT-qPCR assays, and anti-CHIKV IgM. (c) Results from 1 to 21 samples by ORT-snMIRA-LFD assay and RT-qPCR. The experiment was performed in triplicate. NC, negative control.

A total of 329 plasma samples were tested, and 21 were confirmed to be positive for CHIKV. Among the 21 positive specimens, 12 were found to be single positive using RT-qPCR; 4 showed single-positive results for anti-CHIKV IgM; and 5 exhibited both RT-qPCR and IgM positivity (Fig. 6a). Patient information for these 21 clinically confirmed CHIKV-positive specimens is shown in Table S3. Subsequently, the performance of the ORT-snMIRA-LFD assay was assessed using 21 clinically confirmed CHIKV-positive samples and 20 randomly selected negative samples. The results indicated that 18 samples were CHIKV RNA positive, including all 12 RT-qPCR single-positive samples, 5 co-positive samples, and 1 IgM single-positive sample (Fig. 6b; Table S5).

Of the 21 clinically confirmed CHIKV-positive samples, the detection rate of the method established in this study was 85.7% (18 of 21), representing a slight increase over the 81.0% (17 of 21) detection rate of RT-qPCR. In particular, the ORT-snMIRA-LFD assay demonstrated more distinct positive results (evident bands and positive Dr values) for RT-qPCR-detected samples with cycle threshold (Ct) values >35, as exemplified by sample nos. 3–7 (Fig. 6c). These findings indicate that the ORT-snMIRA-LFD assay has better performance in detecting CHIKV RNA than the conventional RT-qPCR method.

## DISCUSSION

The current gold standard for CHIKV detection is one-step RT-qPCR. However, its use in a POCT is limited due to the need for specialized technicians and laboratories. In this study, we introduce a new method called ORT-snMIRA-LFD assay, based on the MIRA technology platform, for the detection of CHIKV (16). The ORT-snMIRA-LFD assay combines the sensitivity of RT-MIRA with the specificity of nested PCR, thereby improving the convenience and effectiveness of the previous methods (13, 16). In this method, the target gene is pre-amplified in the outer RT-MIRA system and then re-amplified in the inner MIRA system, effectively overcoming the amplification plateau and increasing the sensitivity of the method. The use of two primer pairs minimizes non-specific binding and optimizes specificity. A diagram of the ORT-snMIRA-LFD assay is shown in Fig. 1.

Previous studies have used RT-LAMP and RT-RPA for CHIKV detection. In RT-LAMP, fluorescent indicators such as SYBR Green, calcein, hydroxy naphthol blue (HNB), and GelGreen are added to visualize the detection process (17). Previous studies have developed a colorimetric assay of the LAMP reaction using HNB, which allows easy monitoring of CHIKV gene amplification by the naked eye (11, 18). However, it is difficult to determine whether blood samples are positive or negative for CHIKV using the colorimetric method because the color of red blood cells interferes with the HNB color reaction (19). In contrast, the ORT-snMIRA-LFD assay can be visualized using colloidal gold LFD strips, allowing for a more convenient and accurate interpretation of results. In addition, the ORT-snMIRA-LFD assay has a detection sensitivity of up to 1 copy/μL, which is 10-fold higher than CHIKV-specific RT-qPCR. Several previously reported LAMP assays have variable sensitivity (Table S6)(20–23). One study reported an RT-LAMP assay with a sensitivity of 8 PFU/reaction, which is as high as that of conventional RT-PCR (11). However, the ORT-snMIRA-LFD assay developed in this study was subjected to in-house testing and showed superior sensitivity (Fig. 4e).

In addition, the RPA platform has gained significant attention in recent years and has been widely used in POCT for the detection of infectious diseases such as SARS-CoV-2 (24), mpox virus (25), and *Mycobacterium tuberculosis* (26). One study reported the development of an RT-PRA for the detection of CHIKV with a sensitivity of 80 genome copies per reaction, consistent with RT-qPCR (18). Our study showed similar results, with the sensitivity of RT-MIRA being 100 copies/μL, which was 10 times lower than RT-qPCR (Fig. 2g). Several reports in the literature confirmed this result, and to improve the sensitivity of the assay, CRISPR-based technology was introduced into the PRA assay system. Although the performance of the CRISPR/Cas assay is excellent, it serves only as a transduction tool for signal detection and must be used in conjunction with signal amplification technologies such as RPA, ERA, and signal detection tools such as colloidal gold or fluorescence. This results in longer detection times and higher costs (27).

In the realm of traditional RPA technology, the challenge is to increase detection sensitivity while reducing detection costs. To address this challenge, one possible solution is to use nested PCR in conjunction with RPA/MIRA. A recent study developed the snRPA-nfo assay, which uses half-nested PRA to detect *Orientia tsutsugamushi*, and it showed excellent detection performance (28). Our study shows that the ORT-snMIRA-LFD assay is highly analytical sensitive (1 copy/μL) and performs well in detecting clinical samples. Compared to RT-qPCR (81.9%, 17 of 21), ORT-snMIRA-LFD assay had a positive detection rate of 85.7% (18 of 21). Notably, five positive samples with RT-qPCR results had Ct values greater than 36. In most studies, the Ct cutoff for qPCR was 35 (29), and Ct values between 35 and 40 were considered a gray area for qPCR detection. This makes it difficult to define a sample as negative or positive in clinical diagnosis, leading to false-negative results. However, ORT-snMIRA-LFD assay significantly improved the detection accuracy of low-titer samples, showing a clear detection signal on the colloidal gold LFD strip (Fig. 5b). These findings indicate that the ORT-snMIRA-LFD assay has satisfactory performance for the detection of CHIKV RNA and presents the potential for early and expeditious diagnosis of this disease.

Efficient extraction of viral nucleic acid is important to reduce the time required for the ORT-snMIRA-LFD assay. In our study, we attempted to use a rapid nucleic acid release agent for viral RNA extraction. Unfortunately, we did not obtain positive assay results when testing clinical specimens, which we have not presented in this report. While commercial rapid lysis agents have been successful in extracting viral RNA from throat swabs, blood samples have complex matrix effects that affect the efficiency of viral RNA extraction (30). In a similar study, dengue virus RNA was extracted from plasma samples using TNA-Cifer Reagent E, but the results of the clinical sample assay showed sensitivities ranging from 37.5% to 100.0% with unsatisfactory detection performance (31).

Although we were unable to provide an effective method for rapid extraction of viral RNA in our study, we strongly believe that future rapid nucleic acid releasers for blood samples could be used in a way that would significantly reduce the detection time of ORT-snMIRA-LFD assay and make this method quick and convenient to use.

### Conclusion

In this study, we developed a new diagnostic method called the ORT-snMIRA-LFD assay that can detect CHIKV RNA. This technique is highly sensitive, rapid, and user friendly. Our research shows that the ORT-snMIRA-LFD assay is more accurate than the current standard, RT-qPCR. Therefore, it is a promising tool for CHIKV detection.

## SUPPLEMENTAL MATERIAL

Supplemental material jcm.00383-24-s0001.docx Fig. S1 to S3.

Supplemental tables jcm.00383-24-s0002.docx Tables S1 to S6.

ASM does not own the copyrights to Supplemental Material that may be linked to, or accessed through, an article. The authors have granted ASM a non-exclusive, world-wide license to publish the Supplemental Material files. Please contact the corresponding author directly for reuse.
