# Supplementary material for: Rapid and sensitive detection of chikungunya virus using one-tube, reverse transcription, semi-nested multi-enzyme isothermal rapid amplification, and lateral flow dipstick assays
Source: J Clin Microbiol. 2024 Aug 14;62(9):e00383-24. doi: 10.1128/jcm.00383-24 (PMC11389142; doi:10.1128/jcm.00383-24)
Supplement: Supplemental material — Fig. S1 to S3. [file jcm.00383-24-s0001.docx]

**Supplementary Figures**

**
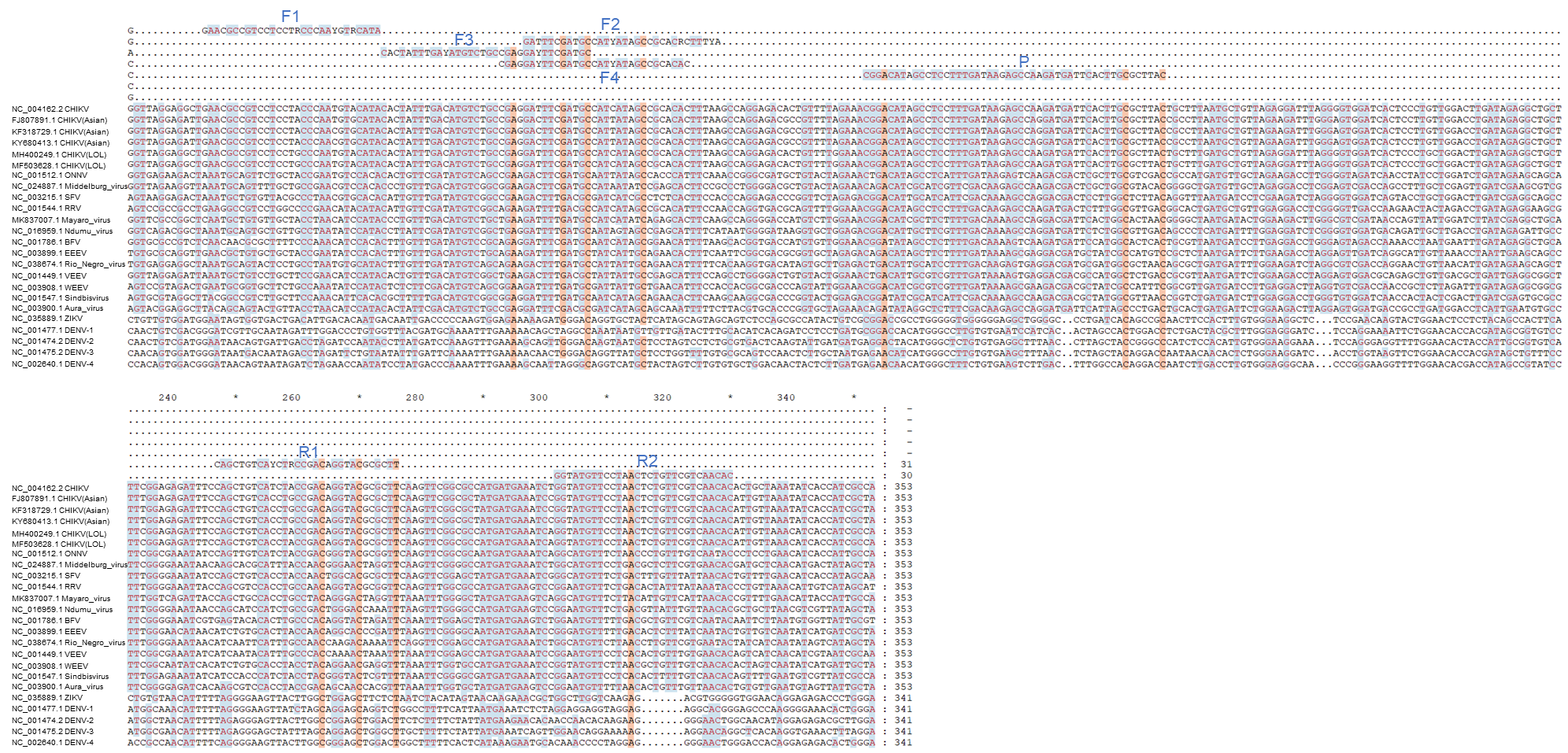
**

**Fig. S1. Positional information of primers and probes on CHIKV NSP4 used in this study**


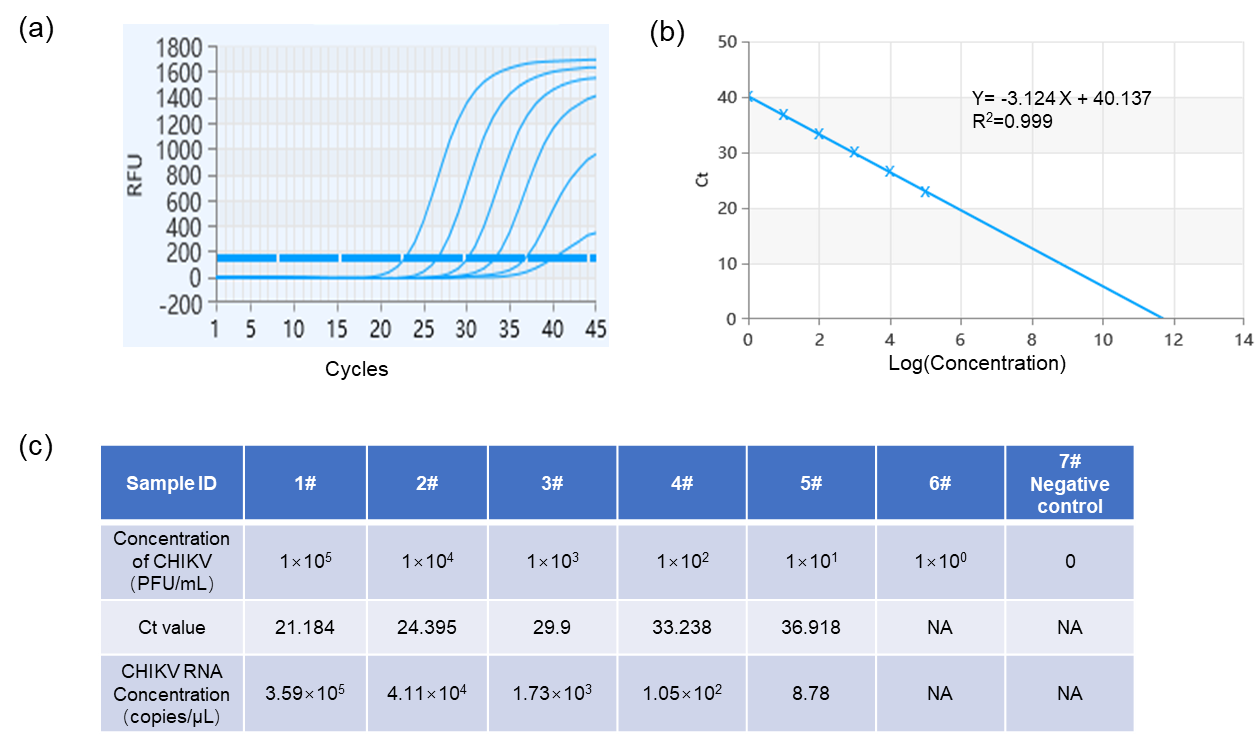


**Fig. S2 Validation of analytical sensitivity for chikungunya virus ORT-snMIRA-LFD**


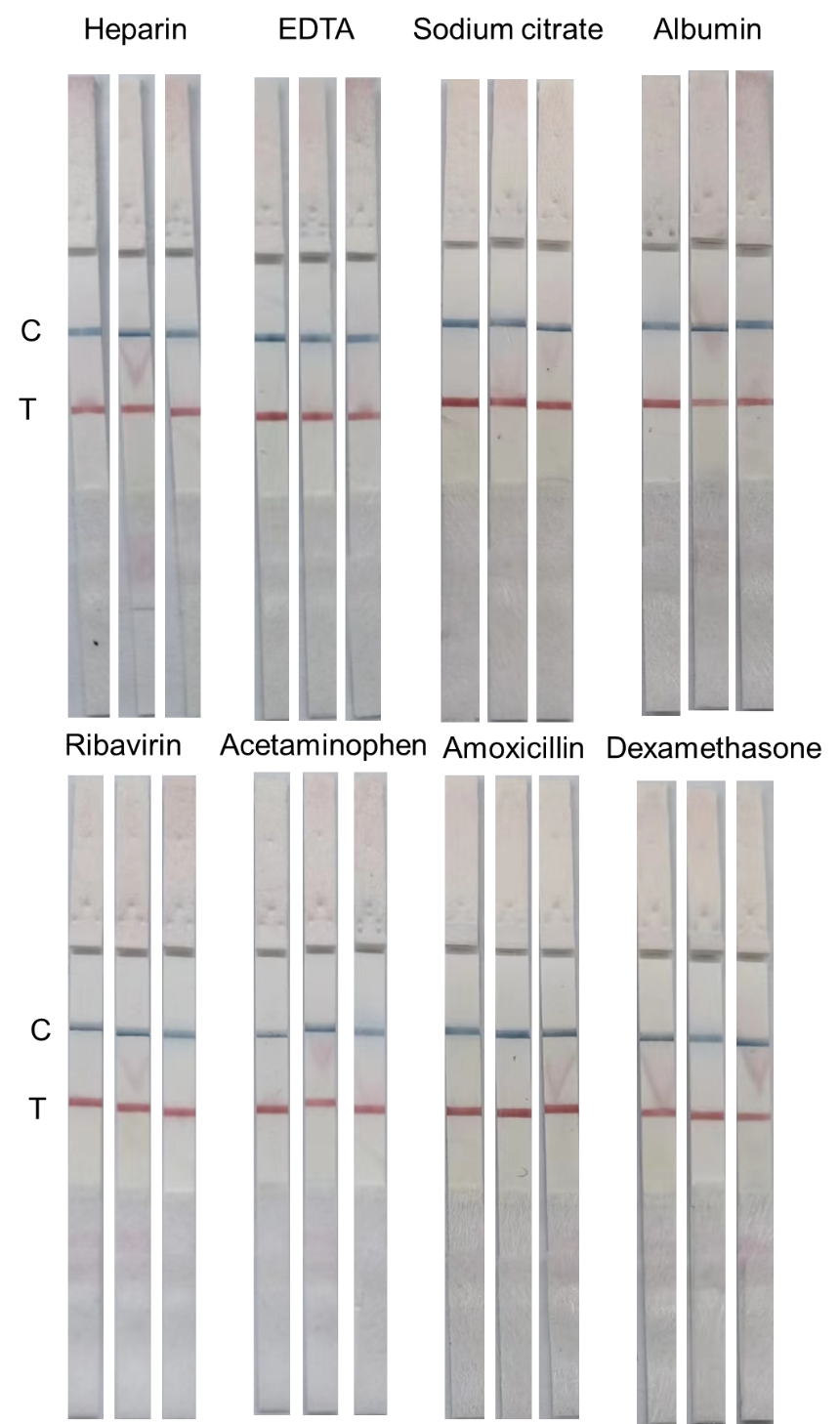


**Fig S3 Results of ORT-snMIRA-LFD anti-interference ability studies**
