# Supplementary material for: Rapid and sensitive detection of chikungunya virus using one-tube, reverse transcription, semi-nested multi-enzyme isothermal rapid amplification, and lateral flow dipstick assays
Source: J Clin Microbiol. 2024 Aug 14;62(9):e00383-24. doi: 10.1128/jcm.00383-24 (PMC11389142; doi:10.1128/jcm.00383-24)
Supplement: Supplemental tables — Tables S1 to S6. [file jcm.00383-24-s0002.docx]

**Supplementary Tables**

**Table S1 Pathogens used and specificity results for the ORT-snMIRA method.**

| **Species** | **Type** | **Strain/clinical sample** | **Clinical test results** | **Expected signal** | **Obtained signal** |
| --- | --- | --- | --- | --- | --- |
| **Viruses** |  |  |  |  |  |
| Chikungunya virus | RNA | Ross strain (ECSA) | / | + | + |
| Chikungunya virus | RNA | 37997 (West African) | / | + | + |
| Chikungunya virus | RNA | 181-25 (Asian) | / | + | + |
| Zika virus | RNA | GZ-01 | / | - | - |
| Dengue virus serotype 1 | RNA | Clinical isolate  strain | / | - | - |
| Dengue virus serotype 2 | RNA | Clinical isolate  strain | / | - | - |
| Dengue virus serotype 3 | RNA | Clinical isolate  strain | / | - | - |
| Dengue virus  serotype 4 | RNA | Clinical isolate strain | / | - | - |
| Japanese Encephalitis virus | RNA | ATCC SA14-14-2 | / | - | - |
| Ross River virus | RNA | T48/DQ226993 | / | - | - |
| O'nyong-nyong virus | RNA | An D SS 234 | / | - | - |
| Human Immunodeficiency Virus type 1 | RNA | Clinical sample | positive | - | - |
| Hepatitis B virus | DNA | Clinical sample | positive | - | - |
| Hepatitis C virus | RNA | Clinical sample | positive | - | - |
| Cytomegalovirus | DNA | Clinical sample | positive | - | - |
| Adenovirus | DNA | Clinical sample | positive | - | - |
| Epstein-barr virus | DNA | Clinical sample | positive | - | - |
| BK virus | DNA | Clinical sample | positive | - | - |
| **Bacteria** |  |  |  |  |  |
| *Staphylococcus aureus* | DNA | Clinical sample | positive | - | - |
| *Pseudomonas aeruginosa* | DNA | Clinical sample | positive | - | - |
| *Escherichia coli* | DNA | Clinical sample | positive | - | - |
| *Klebsiella pneumoniae* | DNA | Clinical sample | positive | - | - |
| *Acinetobacter baumannii* | DNA | Clinical sample | positive | - | - |
| **Fungi** |  |  |  |  |  |
| *Microsporum gypseum* | DNA | Clinical sample | positive | - | - |
| *Trichophyton rubrum* | DNA | Clinical sample | positive | - | - |
| *Malassezia furfur* | DNA | Clinical sample | positive | - | - |
| *Trichophyton mentagrophytes* | DNA | Clinical sample | positive | - | - |
| **Yeasts** |  |  |  |  |  |
| *Candida albicans* | DNA | Clinical sample | positive | - | - |
| *Candida glabrata* | DNA | Clinical sample | positive | - | - |
| *Candida parapsilosis* | DNA | Clinical sample | positive | - | - |

**Table S2 The primers and probs used for ORT-snMIRA**

| **Primer name** | **Sequence 5′–3′** | **Position in CHIKV RNA sequence*** | **Length (bp)** |
| --- | --- | --- | --- |
| F1 | GAACGCCGTCCTCCTRCCCAAYGTRCATA | 6667-6695 | 29 |
| F2 | GATTTCGATGCCATYATAGCCGCACRCTTTYA | 6719-6750 | 32 |
| F3 | CACTATTTGAYATGTCTGCCGAGGAYTTCGATGC | 6696-6729 | 34 |
| F4 | CGAGGAYTTCGATGCCATYATAGCCGCACAC | 6715-6745 | 31 |
| R1 | Biotin-AAGCGCGTACCTGTCGGYAGRTGACAGCTG | 6901-6930 | 30 |
| R2 | Biotin-GTGTTGACGAACAGAGTTAGGAACATACC | 6956-6984 | 29 |
| P | FAM-CGGACATAGCCTCCTTTGATAAGAGCCAA/idSp/TGATTCACTTGCGCTTAC-C3 Spacer | 6774-6822 | 48 |

*NCBI Reference Sequence: NC_004162.2.

**Table S3 Results of ORT-snMIRA anti-interference ability studies**

| **Interfering substances** | **Concentration of interfering substances (results)** | | |
| --- | --- | --- | --- |
|  | **Low** | **Medium** | **High** |
| Antiviral drug: Ribavirin | 25 μg/mL(+) | 50μg/mL (+) | 100μg/mL (+) |
| Acetaminophen | 1 mg/L (+) | 5mg/L (+) | 10mg/L (+) |
| Antibiotic: amoxicillin | 1 mg/L (+) | 5mg/L (+) | 10mg/L (+) |
| Hormonal drugs: dexamethasone | 5mg/L (+) | 10mg/L (+) | 20mg/L (+) |
| Heparin | 5mg/mL (+) | 15mg/mL (+) | 30mg/mL (+) |
| EDTA | 1μg/mL(+) | 5μg/mL (+) | 10μg/mL (+) |
| Sodium citrate | 0.1 mg/mL (+) | 0.3mg/mL (+) | 0.6mg/mL (+) |
| Albumin | 5 μg/mL (+) | 10 μg/mL (+) | 20 μg/mL (+) |

**Table S4 The results of sample stability assessment**

| **Sample ID** | **Storage**  **Temperature(℃)** | **Storage time(days)** | | |  |
| --- | --- | --- | --- | --- | --- |
|  |  | **1** | **3** | **5** | |
| Sample #1  (1×10^5^PFU/mL) | 4 | +++ | +++ | +++ | |
|  |  | +++ | +++ | +++ | |
|  |  | +++ | +++ | +++ | |
| Sample #2  (1×10^2^ PFU/mL) | 4 | ++ | ++ | ++ | |
|  |  | ++ | ++ | + | |
|  |  | ++ | ++ | ++ | |
| Sample #1  (1×10^5^PFU/mL) | 24 | +++ | +++ | +++ | |
|  |  | +++ | +++ | +++ | |
|  |  | +++ | +++ | +++ | |
| Sample #2  (1×10^2^ PFU/mL) | 24 | ++ | ++ | ++ | |
|  |  | ++ | ++ | ++ | |
|  |  | ++ | ++ | + | |
| Sample #1  (1×10^5^PFU/mL) | -20 | +++ | +++ | +++ | |
|  |  | +++ | +++ | +++ | |
|  |  | +++ | +++ | +++ | |
| Sample #2  (1×10^2^ PFU/mL) | -20 | ++ | ++ | ++ | |
|  |  | ++ | ++ | ++ | |
|  |  | ++ | ++ | ++ | |

Note: Dr>1.0 indicates strong positive (+++), 0.7<Dr<1.0 indicates medium positive (++), 0.2<Dr<0.5 indicates weak positive (+), and Dr<0.2 indicates negative.

**Table S5 Basic clinical data and storage parameters for CHIKV clinical specimens**

| **ID** | **Sex** | **Age** | **Symptoms at time of visit** | **Time of onset of illness** | **Sample type** | **Conditions of specimen storage** | **Storage time** | **Freezing frequency** | **RT-qPCR** | **IgM** | **Diagnostic** | **ORT-snMIRA-LFD** |
| --- | --- | --- | --- | --- | --- | --- | --- | --- | --- | --- | --- | --- |
| YCV01 | F | 25 | Fever (38.9°C), muscle pain, rash | 1 day | Plasma | Store at 2-8°C for 1 day, then transfer to -20°C | 24 days | 4 times | Negative | Positive | Positive | Positive |
| YCV02 | M | 49 | Fever (38.2°C), cough | 2 days | Plasma | Store at 2-8°C for 2 days, then transfer to -20°C | 21 days | 4 times | Negative | Positive | Positive | Negative |
| YCV03 | M | 43 | Fever (38.9°C), cough | 1 day | Plasma | Store at 2-8°C for 1 day, then transfer to -20°C | 22 days | 4 times | Positive | Negative | Positive | Positive |
| YCV04 | M | 22 | Fever (39.3°C), headache, chills | 4 days | Plasma | Store at 2-8°C for 1 day, then transfer to -20°C | 16 days | 4 times | Positive | Negative | Positive | Positive |
| YCV05 | M | 24 | Fever (39.1°C), cough | 2 days | Plasma | Store at 2-8°C for 1 day, then transfer to -20°C | 19 days | 4 times | Positive | Negative | Positive | Positive |
| YCV06 | M | 43 | Fever (39.2°C), headache, muscle pain | 2 days | Plasma | Store at 2-8°C for 3 days, then transfer to -20°C | 21 days | 4 times | Positive | Negative | Positive | Positive |
| YCV07 | M | 24 | Fever (39.0C), chills, headache, vomit | 3 days | Plasma | Store at 2-8°C for 1 day, then transfer to -20°C | 18 days | 4 times | Positive | Negative | Positive | Positive |
| YCV08 | M | 35 | Fever (38.6°C), cough | 0 day | Plasma | Store at 2-8°C for 1 day, then transfer to -20°C | 15 days | 3 times | Negative | Positive | Positive | Negative |
| YCV09 | M | 34 | Fever (38.8°C), cough | 4 days | Plasma | Store at 2-8°C for 1 day, then transfer to -20°C | 14 days | 3 times | Negative | Positive | Positive | Negative |
| YCV10 | M | 43 | Fever (38.8°C), cough | 2 days | Plasma | Store at 2-8°C for 1 day, then transfer to -20°C | 14 days | 3 times | Positive | Negative | Positive | Positive |
| YCV11 | M | 56 | Fever (38.9°C), muscle pain | 3 days | Plasma | Store at 2-8°C for 3 days, then transfer to -20°C | 12 days | 3 times | Positive | Negative | Positive | Positive |
| YCV12 | M | 34 | Fever (38.2°C), muscle pain, rash | 0 days | Plasma | Store at 2-8°C for 1 day, then transfer to -20°C | 21 days | 3 times | Positive | Positive | Positive | Positive |
| YCV13 | F | 38 | Fever (38.6°C), muscle pain | 3 days | Plasma | Store at 2-8°C for 3 days, then transfer to -20°C | 22 days | 3 times | Positive | Negative | Positive | Positive |
| YCV14 | M | 37 | Fever (38.0°C), cough | 4 days | Plasma | Store at 2-8°C for 2 days, then transfer to -20°C | 16 days | 3 times | Positive | Positive | Positive | Positive |
| YCV15 | M | 49 | Fever (38.3°C), headache | 2 days | Plasma | Store at 2-8°C for 1 day, then transfer to -20°C | 15 days | 1 time | Positive | Negative | Positive | Positive |
| YCV16 | F | 55 | Fever (37.9°C), muscle pain, rash | 1 day | Plasma | Store at 2-8°C for 1 day, then transfer to -20°C | 15 days | 1 time | Positive | Negative | Positive | Positive |
| YCV17 | M | 34 | Fever (38.5°C), muscle pain, rash | 2 days | Plasma | Store at 2-8°C for 3 days, then transfer to -20°C | 3 days | 1 time | Positive | Positive | Positive | Positive |
| YCV18 | M | 28 | Fever (38.2°C), cough | 3 days | Plasma | Store at 2-8°C for 3 days, then transfer to -20°C | 3 days | 1 time | Positive | Negative | Positive | Positive |
| YCV19 | M | 37 | Fever (38.7°C), muscle pain, headache | 1 day | Plasma | Store at 2-8°C for 3 days, then transfer to -20°C | 3 days | 1 time | Positive | Positive | Positive | Positive |
| YCV20 | M | 41 | Fever (38.8°C), muscle pain, rash | 2 days | Plasma | Store at 2-8°C for 3 days, then transfer to -20°C | 3 days | 1 time | Positive | Negative | Positive | Positive |
| YCV21 | F | 29 | Fever (38.6°C), muscle pain, rash | 2 days | Plasma | Store at 2-8°C for 3 days, then transfer to -20°C | 3 days | 1 time | Positive | Positive | Positive | Positive |

**Table S6 Comparison of CHIKV detection protocols used in the current and previous studies**

| Methods | Target | Sample type | Number of clinical samples | Lowest limit of detection | sensitivity /specificity | Reference |
| --- | --- | --- | --- | --- | --- | --- |
| Real time qPCR | E1 | Serum | 37 | 3.95 RNA copies/reaction | 76%/100% | (10) |
|  | NSP1 | Serum | 10 | 0.5 PFU/mL | n.a. | (29) |
|  | NSP1 or E1 | Serum | n.a. | 5 copies/μL or 50 copies/μL | n.a. | (21) |
|  | 3′ UTR | Serum | 40 | 60 RNA copies/reaction | 100%/100% | (22) |
| RT-LAMP | E1 | Serum | 42 | 50 pfu/reaction | RNA sample :70%, serum samples:58%/100% | (23) |
|  | E1 | Serum | 10 | 8 pfu/reaction | n.a. | (19) |
|  | 3′ UTR | Serum | 37 | 66 RNA copies/reaction | 80.43%/100% | (20) |
|  | 6K-E1 | Serum | 35 | 163copies/reaction | 100%/80% | (11) |
| RT-RPA | NSP1 | Serum | 58 | 80 genome copies/reaction | 100%/100% | (18) |
| **ORT-snMIRA** | **NSP4** | **Plasma** | **21** | **1 copies/**μL | **na** | **This study** |
